# Oral health perceptions and practices of caregivers at children’s religious schools and foster care centers: a qualitative exploratory study in Lahore, Pakistan

**DOI:** 10.1186/s12903-022-02687-0

**Published:** 2022-12-24

**Authors:** Javeria Saleem, Muhammad Ishaq, Muhammad Salman Butt, Rubeena Zakar, Ushna Malik, Maida Iqbal, Florian Fischer

**Affiliations:** 1 Department of Public Health, Institute of Social and Cultural Studies, University of the Punjab, Lahore, Pakistan; 2 Department of Sociology, Institute of Social and Cultural Studies, University of the Punjab, Lahore, Pakistan; 3 Department of Public Health, University of the Punjab, Lahore, Pakistan; 4 Lahore Medical & Dental College, Lahore, Pakistan; 5 Institute of Public Health, Charité–Universitätsmedizin Berlin, Berlin, Germany; 6 Bavarian Research Center for Digital Health and Social Care, Kempten University of Applied Sciences, Kempten, Germany

**Keywords:** Pediatric dentistry, Health awareness, Oral health, Dental health perception

## Abstract

**Background:**

Oral diseases are one of the major public health problems worldwide and affect the population of all age groups. This qualitative study aimed to explore the perceptions and practices of caregivers at care centres and boarding religious schools responsible for managing children’s oral health.

**Methods:**

A qualitative ethnomethodological approach was used to collect data from the caregivers at the children’s religious schools and foster care centres. A purposive sampling technique was used to conduct focus group discussions comprising 4–7 caregivers from five foster care centres and religious schools located in Lahore, Pakistan.

An interview guide was developed based on results from previous studies. An inductive approach was used to analyse data on broader oral health concepts to generate themes in this qualitative research. A three-step thematic analysis was applied to develop codes that were merged to generate categories and to conclude into themes from the transcribed data. Five focus group discussions were conducted at two foster care centres (FG1 & FG2) and three religious schools (FG3, FG4 & FG5). Foster care centres had children of both gender within the same premises; however, religious schools had segregated settings.

**Results:**

The following four themes emerged from the thematic analysis: development of the desired living environment and responsibilities of the caregivers, preexisting traditional personal knowledge of the caregivers determine children’s oral health, use of religio-cultural driven and convenience-based oral hygiene practices, and ethnomedicine, spiritual healing, and self-medication. Development and the existing living environment of the foster care centres and religious schools appeared important to manage the matters of the boarding children.

**Conclusions:**

This qualitative study concludes that the oral health of the children at foster care centres and at religious schools depends upon the personal reasoning and pre-existing religio-cultural knowledge of the caregivers rather than on specialized oral health-oriented approaches. The foster care centres are more involved in supervising the children to maintain oral hygiene and oral health compared to religious schools.

**Supplementary Information:**

The online version contains supplementary material available at 10.1186/s12903-022-02687-0.

## Background

Oral diseases are one of the major public health problems worldwide and affect the population of all age groups [1]. Oral health is defined as standardized health of the mouth and being free from any chronic pain of the mouth and adjacent tissues that inhibits an individual from speaking, smiling, eating, and socializing with any uncomfortable feeling of active disease [2]. Previous studies have provided evidence that poor oral health can cause further health problems [3], and the healthy development of a child can be recognized by good oral health [4].

According to the USA Center for Disease Control, the incidence of tooth decay is four times more common among adolescents aged 14–17 years than asthma [5]. The role of parents is vital in establishing and sustaining children’s oral health behaviour. The belief of parents, role modelling, parenting self-efficacy, and parenting style are key determinants along with the social gradients to define the oral health of a child [6–8].

Children living in foster care centres suffer from poor physical, emotional, and oral health problems as they live in normal family settings [9]. Several studies have highlighted the health needs of children at foster care centres, but limited evidence has been reported on oral health outcomes. The American Academy of Pediatrics reported that approximately 54% of the children and teens who enter foster care centres have considerable dental and oral health problems [10].

Children are being placed in foster care centres for multiple reasons, but poverty and food security in low socioeconomic countries such as Pakistan are prime concerns [11]. Other than the formal foster care centres in Pakistan, “Madrasa” (Religious Schools) is the widely acceptable choice for orphans and poor children [12]. Some religious schools are well equipped with modern necessities, including hostels and foster care facilities for children. The pattern of care for children in these religious schools is the same as that of formal foster care centres, and the caregiver is called “Nazim” (administrator).

The majority of the children enrolled in foster care centres are poor orphans and are neglected by their society [11]. Enrollment in religious schools also has a strong religious reason. Enrollment figures showed that approximately two million children are enrolled in 22,000 religious schools in Pakistan. This figure is not substantiated to be publicly verified but can be estimated from the data of the Pakistan household survey 2001 [13].

Despite the significance of oral health, dental care assessment and data reporting and management related to children's oral health remain limited [10]. The scarcity of updated data on children's oral health is also evident in Pakistan considering the perceptions of caregivers [14]. Evidence has suggested that caregivers at children’s care centres require a better understanding of oral health to improve children's oral health. An ethnomethodological qualitative research design can help to explore the complex oral health issues in children's foster care centres. Until now, the oral health of children living in foster care centres and religious schools in Pakistan has not been considered a serious health issue in previous studies. Therefore, this qualitative study aims to explore the perceptions and practices of caregivers in Lahore, Pakistan, at the children's foster care centres and religious schools in managing children’s oral health. This study is novel, and the findings will help to provide evidence for improving oral health interventions for children by enhancing the understanding of caregivers’ roles.

## Materials and methods

### Research team and reflexivity

Consolidated criteria for reporting a qualitative research checklist (COREQ) were used to report the findings of this qualitative research [15]. The research team which planned and conducted the focus group discussions, having a background in public health, social sciences (PhD), and dentistry (BDS), had 5–10 years of experience in research and clinical dental health practices.

Government-registered (≥ 10 years) not-for-profit foster care centres and religious schools situated in Lahore, Pakistan, were selected. Inclusion criteria of foster care centres and religious schools were having at least 50 children enrolled, having hostel facilities, and supervising children the carers. The research team planned the field visits to the selected foster care centres and religious schools to formally introduce themselves and establish a conducive environment to collect data from the caregivers. A summary of the study with the researchers’ biography was given to the concerned personnel to be shared with the caregivers. The research team also briefed the administration of the foster care centres and religious schools on the objectives of this research and sought formal permission to collect the data from caregivers. The researchers’ affiliation with the public health and social sciences department of the government sector university helped to seek the required permissions. The selected foster care centres and the religious school’s administration were requested to schedule the focus group discussion (FGD) within the next 2–3 days.

### Study design

The theoretical roots of this study are based on the ethnomethodological approach and focused on oral health practices of the caregivers at foster care centres and religious schools. The ethnomethodological approach provides the tool to understand the everyday practices of the caregivers. Most of the foster care centres and religious schools in Lahore, Pakistan, have scarce resources. This study explored oral health practices and oral health hygiene by conducting FGDs. These FGDs and ethnomethodology provided the freedom for the researchers to focus on a detailed formal analysis of everyday oral health practices. This exploratory study design allowed to explore the caregivers’ perceptions, beliefs, and practices related to oral health while managing children’s oral health.

### Sampling and participants

The caregivers were nominated by the foster care centres’ and religious schools’ administration to participate in the FGDs, comprising 4–7 participants, by using a purposive sampling technique. Here, the participants had two to three days time to decide wehter they wold like to take part in the FGDs. The caregivers of both sexes should be between 18 and 50 years of age and should have an experience of ≥ 5 years in taking care of children (0–16 years). The FGDs began with the introduction of the researchers explaining their institutional affiliations, qualifications, and reasons for conducting this research to build confidence and trust. Verbal informed consent was obtained from all the participants to become a part of this audio-recorded face-to-face FGD. The selected participants did neither refuse nor withdraw consent, did not drop out for any reason, and completed the entire duration of FGDs. The data were collected until a saturation point was achieved where no additional information was received and researchers covered all the questions from the interview guide (see Additional file 1).

### Setting

The face-to-face FGDs were conducted in the conference rooms of the selected centres to avoid any interruption. The centres without conference rooms arranged separate rooms for FGDs to facilitate the researchers. The FGDs were conducted with the selected participants, and the presence of any additional personnel was avoided by labelling the entrance door. The FGDs were audio recorded. The caregivers’ demographic characteristics were also recorded, and the discussion was driven by the researchers following a semistructured interview guide.

### Data collection

Five FGDs (one from each foster care centre/religious school) were conducted during the autumn of 2020 to explore the participants’ perceptions and practices regarding oral health. A semistructured focus group discussion guide was developed based on previous studies [9, 16]. The interviews were conducted in the Urdu language and transcribed into English dialects for data analysis. The field notes were collected during the interview to record the participants’ important gestures. Debriefing with the study participants and among the research team themselves was a continuous process throughout the fieldwork that helped to clarify the concepts and endorse the extracted meanings.

An inductive-deductive approach was utilized to collect data on oral health perceptions and practices to extract the in-depth information narrated by the caregivers by conducting focus group discussions. The deductive approach was utilized to develop the study guide by using verified reasoning published in previous studies [9, 16]. Furthermore, the interview guide was pilot tested to ensure that the questions were correctly worded and understandable to extract the relevant information. The inductive approach was used to analyse data on oral health concepts to generate themes in this qualitative research.

Trustworthiness and procedural consistency were formed to ensure the validity and reliability of the study findings by ‘*Prolonged engagement’* and ‘*Persistent observation’* and tape recording of the interview. Credibility was attained by peer-checking of the interview statements in the data and their relevance in the results. Transferability was determined by a full description of the research context and analytical generalization.

### Data analysis

**Fig. 1:**
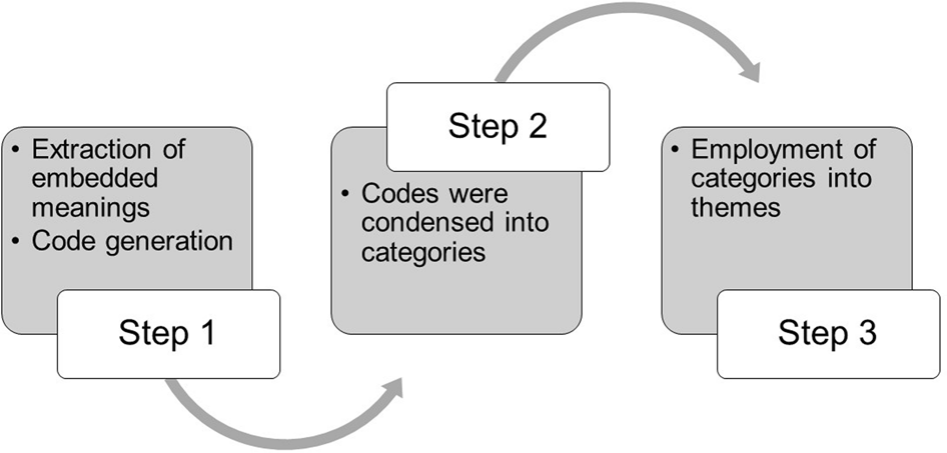
Flowchart explaining the data analysis procedure

The FGDs were 45–90 min in duration, and all were audio-recorded at the foster care centres and religious schools by a moderator and a recorder. Verbatim transcribing of focus group audio recordings was performed by the research team for a three-step tandem data analysis. The data were coded by the authors (JS, MIs, MS, and RB) who had expertise in conducting qualitative research. Meaningful statements consistent with the perceptions and practices of study participants were repeatedly examined and coded (Step 1) using the Open Code software v 4.03. The identified codes were classified to generate the categories (Step 2). Research themes were employed using the categories to extract the in-depth information narrated by the caregivers about the oral health phenomenon (Step 3), as shown in Fig. 1 [17].

### Ethical considerations

The institutional review board of the University of the Punjab approval was sought before the field data collection (Ref #218/D FEMS). Informed consent was obtained from the heads of the children's care centres and individual participants. The anonymity of the children's foster care centres, religious schools and focus groups was assured by enumerating them with a fictitious number (FG1 to 5). All audio recordings and transcripts were stored on a secure electronic device and were password protected.

## Results

Five children’s care centres located in Lahore, Punjab (two foster care homes and three religious schools) were purposively selected to ensure the diversity and inclusivity of the data using a criterion sampling technique. These care centres belong to the public and private sectors and have different socioeconomic, cultural, and administrative settings. Inhabited children were from different geographic, ethnic, socioeconomic, and educational backgrounds. The foster care centres had a capacity of 74–250 inhabitants, and the age group varied for religious schools (7–19 years) and foster care centres (newborn to 18 years).

Five focus group discussions were conducted at two foster care centres (FG1 & FG2) and three at religious schools (FG3, FG4 & FG5). The inclusion of both foster care homes and religious schools allowed the researchers to compare the oral health practices between formal and religiously oriented settings. A focus group of 4 to 7 caregivers was formed. The age range of the caregivers was from 23 to 50 years for both males and females. At religious schools, the caregivers were teaching children in a formal school setting and also monitoring their daily life activities in the hostels. Foster care centres and religious school hostels had quite different environments and responsibilities, as the age group of the children varied in the two settings. In the foster care centres, the ages of the children under care ranged from 0 to 14 years; however, in the religious schools, the ages ranged from 8 to 16 years. In both settings, caregivers and the hostel staff were responsible for the children, where most of them were orphans and poor except in FG3, where most of the children were from the high socioeconomic class.

Foster care centres had children of both genders within the same premises; however, religious schools had segregated settings. For example, the children’s care centres were further divided into different categories based on the facilities available, as shown in Table 1.

**Table 1:** Characteristics of study participants

Focus group	Children center	Children inhabited	Availability of facilities	Number of participants in the focus group	Composition of the focus group
FG1	Foster Care home	Both genders	Moderate	Six	Females only
FG2	Foster Care home	Both genders	Moderate	Seven	Females only
FG3	Madrasa	Boys only	High	Five	Males only
FG4	Madrasa	Girls only	Low	Six	One male, five females
FG5	Madrasa	Boys only	Low	Four	Males only

**Fig. 2:**
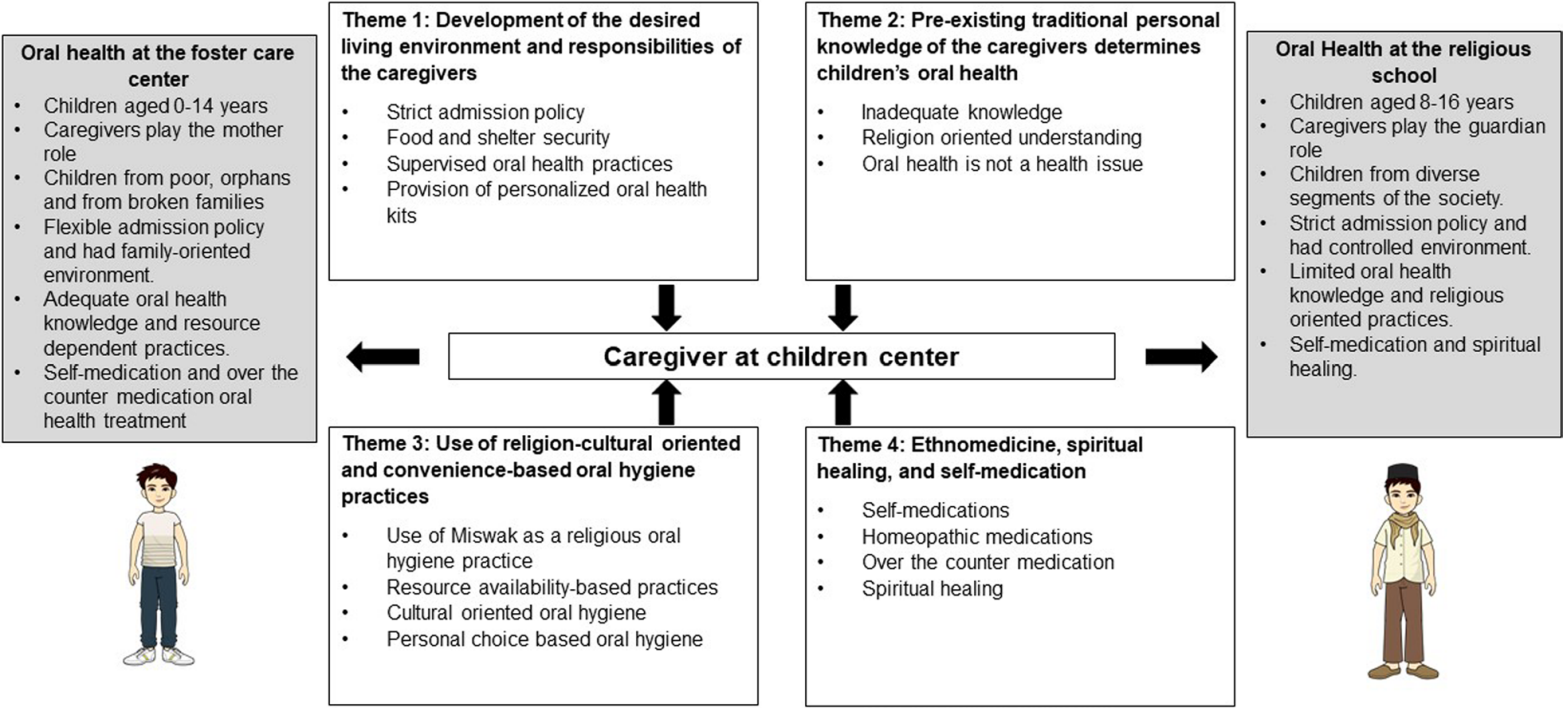
Summary of the results and comparison between foster care center and religious school oral health

The following four themes emerged from the thematic analysis: development of the desired living environment and responsibilities of the caregivers, preexisting traditional personal knowledge of the caregivers determine children’s oral health, use of religio-cultural driven and convenience-based oral hygiene practices, and ethnomedicine, spiritual healing, and self-medication. These themes were supported by the extracted categories, as shown in Fig. 2.

### Development of the desired living environment and responsibilities of the caregivers

This is an implicit emergent theme that appeared important to determine the importance of oral hygiene and health and the role and responsibilities the caregivers play to maintain oral hygiene and health of the children under care.

This theme principally demonstrates the demographic internal environment and responsibilities of the caregivers at the foster care centres and religious schools. In the foster care centres, each caregiver was allocated 6–11 children as a mother. However, in the religious school, the caregiving responsibilities were different and split between teachers and hostel wardens. Hostel wardens were mainly responsible for the health issues of the children, including dental issues, whereas the teachers were responsible for checking the overall cleanliness of the morning assembly.

The researchers found the living environment important at these children's centres which determined the overall affairs of the children including their oral health. One of the caregivers, while narrating their admission policy:

FG3: *“We have the policy to keep children under observation before finalizing the admission, the reason is to admit more polite and civilized children, such as they don’t use slang language, they don’t smoke and they respect the elders. This all helps to maintain the hostel environmentally friendly and inclusive for learning and living.”*

It appeared that the religious schools had a preset policy to enroll children with good moral conduct. These children were evaluated and assessed for their family background and parents’ recommendations at the time of enrollment. Well-mannered and obedient children make it easier for caregivers to control and maintain the institutional environment. The possible reason for this policy could be that most of the children enrolled were mature. However, the foster care centres had no such policy, and they had the obligation to enroll all the deserving children to accommodate and train them.

FG1: *“They come from different parts of Punjab; we offer them school fees, books, and everything they need, and they stay with us up until the 10*^*th*^* level. However, in the majority of cases, they come in bad conditions both physically and mentally, including attitudes, and frightened, but we understand that they come from broken families. It takes time for them to adjust and for us to train them to live in the new settings, especially those coming from the villages, it becomes more difficult for them to adjust.”*

When probed further about the dental conditions at the time of enrollment, the answer was that:

FG1: “*I will say very bad, we have to teach them even how to hold the brush, at the age of 8/10.”*

The result reflected that a limited focus is being made on maintaining the good oral hygiene of these children. It was a matter of great interest to unleash how the foster care staff motivates children to oral hygiene in their environment.

FG2: *“We have a bucket of toothbrushes and paste in it which we take to the dining room after every child has finished his/her eating we ask them to clean their teeth and at night we don’t let them eat anything after that.”*

Another one added when asked if they ask them to brush:

FG2: *“They will not do, they throw toothpaste into the sink if one of us is not with them.”*

FG4: *“We feel really difficult to communicate with new girls; they have a village lifestyle, low understanding, and communication level, which takes time to adjust. However, with time they start copying their bajis (elder sisters or seniors) in the hostel, which includes tooth brushing with other cleanliness routines. Sometimes we ask our donors to help us buy separate small bags or kits to keep their soaps, shampoos, and other such stuff because we live in condensed settings with 10 to 12 girls in a room.”*

These girls were given education on maintaining cleanliness, including oral hygiene, until adolescence by caregivers. These girls also tried to imitate their senior girls to maintain and practise their oral health and hygiene. Oral health at these care centres mainly relies on donations and funds. The scarcity of resources can easily affect the normal oral health practices and hygiene of these girls.

### Preexisting traditional personal knowledge of the caregivers determines children’s oral health

The importance of oral health knowledge has been cited in several previous studies that developed our interest in exploring this phenomenon concerning caregivers at children’s care centres. The researchers were keen to explore the knowledge and perceptions of the caregivers about the oral health that they were utilizing to manage oral hygiene and oral health at the centres, and diverse narratives and understandings emerged from the data.

FG4: “*To be honest we did not take it as a health thing (making gestures with the index and middle finger) it’s nice to interact with you we can learn something from you. However, we consider it routine cleanliness. We expect our girls to brush in the morning before class to avoid mouth smell… No, I have not seen anyone doing it at night, but it seems it’s important at night too.”*

The group appeared keen to learn about the importance of oral care and health as a “*health matter*”, which did not appear anywhere on their priority list before.

FG5: “*It’s a Sunnah of the Holy Prophet, science is telling these things now but our Prophet told us 1400 years ago, we use miswak 5 times a day before every prayer, by doing this we get sawab (merits), but on the other hand, its cleanliness and has health benefits too. Although newcomers to our madrasah don’t know much about this, with time they get used to it. Children tease each other if someone’s mouth is smelling. If someone has not brushed or done miswak, teachers can determine when they are reading in front of the teachers… Yes, but very rarely, that we ask someone to go out of the class and come back after brushing.”*

Religious schools considered oral hygiene as a religious virtue and an act of Sunnah, which makes Miswak an esteemed practice at these children's care centres.

FG3: “*We do organize health camps from time to time, but I don’t think we ever had a precise focus on oral health, we only consider this when we have such issues as toothache. I think one reason is that our kids go home every weekend, so their parents manage their health issues. Recently, we realized that those kids use… This toothpaste has more oral health issues than those that use expensive or imported brands. As my colleague said, children who come from well-off backgrounds do not have many oral health issues, but we are providing good toothpaste to orphans and poor ones.”*

The caregivers at religious schools with high facilities appeared well-educated and aware of oral health and oral hygiene practices. However, children’s health matters were mainly managed by their parents. Oral health seems to be a priority for the well-off segment of society rather than for the poor. This can also be observed in the following statement by a less privileged religious school.

FG4: *“I was thinking during this whole time that why we don’t know much about this or consider it as an issue, actually we eat simple food, so our kids’ teeth don’t get that much spoiled, we don’t smoke or chew chalya* (betle) *it is considered a bad practice among the religious people.”*

Similar views were presented by another group:

FG3: *“You doctor people are taking it as a health issue but if I can tell you about my understanding, in society we just think our mouth should not stink, it causes shame and if someone speaks up then it can create serious embarrassment for the person.”*

The caregivers at the religious schools had the perception that oral health is not a health issue which requires medical consultations unless they do not have any bad mouth odour. They also considered it to be doctors’ matter who projected it as a health issue to maintain oral health and hygiene.

However, the foster care centre staff had a different opinion about the oral health practices at their centres.

FG1: *“We have instructions from our madam if anyone has any health issue should consult a doctor immediately. We don’t hear much about dental health but cleanliness among children. Some of our colleagues have suffered from oral health issues, but I don’t think children can have any dental problems at this age.”*

This shows their lack of understanding that children can not suffer from oral health issues because they are very young to have such problems and that oral health issues are only for the adult or older segments of society.

### Use of religio-cultural driven and convenience-based oral hygiene practices

Apart from using different brands of toothpaste, it appeared that people use several methods for oral hygiene. These practices have regional and cultural variations. In religious settings, it appeared that *miswak* or chewing sticks were popular as a part of religious practices. Focus groups 1, 2, and 4 were not using this for their children, but participants themselves were using it also with *dantan* or walnut skin.

FG5: “*As my colleague said we consider miswak Sunnah* (the practice of the Holy Prophet) *and use it 5 times for sawab,*(reward) *but our kids also use toothpaste at night and in the morning.”*

For FG5 oral hygiene practice was mainly a religious matter, however, children use it sometimes but it appeared they were not supervised strictly to do so. One of the foster care centres narrated their experience in the following statement:

FG2: “W*e don’t use any specific kinds of toothpaste, it depends on what our donors give us, we keep one toothpaste in the bucket of the toothbrushes.”* Because they mentioned that sometimes their 2- to 3-year-old children also brush their teeth to imitate the older ones. When probed if there is any different toothpaste for them, they stated, “*No, we use only one for everyone.”* When probed, another participant said, *“I didn’t know if there are any special kinds of toothpaste for the smaller kids.”*

The use of *dantan* (walnut skin) by some of the children was narrated by some of the participants:

FG5: *“We have some Kashmiri students who use dantan, it cleans very well but you can use it only once or twice a week”* when asked about the reason, that why it cannot be used, everyone started laughing, a senior one replied, *“everyone is laughing because it is considered a girlish thing, it whitens the teeth but your lips and gums become like you have used a red lipstick”*. Another offered his input: *“There is a serious thing about it; although dantan soothes teeth and gums if you use it for longer it damages gums and exposes the roots of the teeth.”*

It shows that the children at the religious schools use different oral hygiene products based on their own socio-cultural understandings no matter if they are harmful to their oral health*.* These practices are not linked with the children at the religious schools only but the caregivers at the fosters care centres appeared using personal reasonings for the children’s oral health.

FG2: *“In these days due to corona (COVID-19 Pandemic) we are using black seeds water, we give it to our kids in the morning, we think it helps oral health too, it’s bitter taste is also good for the teeth”* another gave her input that; “*it reminds me now, we have not seen any complaints about the teeth in these days, it means it’s good for the teeth too, however, it kills stomach worms if anyone has, you know these things are common among the children.”*

Oral health is a personal matter that varies from culture to culture and from area to area. Hence, personal knowledge or information these people receive also matters in their choices of oral hygiene practices.

Another phenomenon we wanted to explore was the use of the toothbrush. We asked about the duration of time that the children used one brush.

FG2: “*I think it lasts for 6 months; after six months, we ask our donors to provide us with new brushes. No, we change for everyone together, you know if we change for some and not for others. They will feel bad that some have got new brushes, you know kids are kids and we don’t want to give an impression of discrimination.”*

FG1 informed that they replaced the manual toothbrush after a couple of months:

“*I think we change every 2/3 months, my colleague can confirm, yes, she is right we change after every 3 months, if someone breaks or it falls at some dirty place then we can give a new one.”*

This shows the lack of seriousness towards oral hygiene by using a toothbrush for that long and its durability for effective cleanliness.

Except for FG3, no one appeared concerned about the selection of the toothpaste brands. FG3 described that they discourage the use of one specific local brand, and they observed that they received more complaints about oral health or toothache. However, all others were using that specific brand frequently even saying the toothpaste they were using was the name of the brand.

### Ethnomedicine, spiritual healing, and self-medication

Toothache appeared a common problem both at the foster care centres and religious schools; in the beginning, when asked about this, the caregivers answered the question negatively, but later they shared their experiences of the treatments and techniques they used. Diverse treatment methods and approaches appeared from group to group, ranging from allopathy, traditional and spiritual to homeopathic treatment.

FG1: *“When someone complains about toothache, we start with Panadol, but if it doesn’t work then we take the child to the hospital, but doctors cannot do much for the small children, they cannot extract the tooth out”.* When asked about the use of traditional treatment, *“madam has instructed us not to use any totkay (tricks).”*

They tried to show that they consider dental aches and treatment serious, FG2 described their practices in the following statement:

FG2: “We *have a tray of medicine, our doctor provides us with a very effective homeopathic medicine for toothache though it has a very strange taste, we put it on cotton and place it on the aching tooth.”*

On many occasions, it appeared that there was no unified oral health guidance, but personal preferences and availability of the options mattered for the caregivers.

It appeared that hardly anyone will go to the dentist for oral health, mainly physicians or, in some cases, first aid providers, prescribe the treatment. Even personal contacts appeared important to refer to someone.

FG5: “*There are many medical stores around us we know them personally.”* When asked if they know about a person’s speciality they told: “*We never asked them but as long as they provide medicines and they are effective, this is fine.”*

Caregivers at the girl’s madrasa appeared more using spiritual healing methods:

FG4: “*I learned some methods during my studies that are very effective for toothache, I tell my students to practice those, there are some verses from the Holy Quran and supplication, another one is very effective”.* When asked do girls give feedback about its effectiveness: “*Those who have a strong belief in this, it gives relief, but if someone doubts it, it will not work for them.”*

This is not surprising for a traditional society such as Pakistan, where there are quakes those claim to treat every health condition from toothache to cancer and the very recent COVID-19 pandemic. They are sitting in the markets for years and claiming to treat every illness.

## Discussion

A summary of this study's results is depicted in Fig. 1. The overarching findings of this study highlight several fundamental aspects related to the caregiver’s perceptions and practices at the foster care centres and religious schools in Lahore. Food and shelter security at children’s care centres is one of the major reasons for poor and orphan children. The enrollment policy of the foster care centres was found to be comparatively flexible to that of the religious schools considering the significant age difference. Madrasa children appeared to be less supervised of their health issues than those of the foster care centres.

The role of caregivers was found to be important for developing a healthy environment for the children for a living and their oral health. A nurturing, controlled and the conducive environment was observed at the foster care centres with a stringent induction policy. However, centres and religious schools with no such policy found it difficult to maintain a healthy living environment. The consistency of behavioural habits also depends upon the practices of the caregivers [7]. The role of the caregiver is one of the fundamental principles of social determinants of health [18]. Where the caregiver lacked adequate knowledge about oral health and not supervising the children, the children were using poor techniques for oral hygiene. An imitating environment was also observed in one of the children’s care centres where the children followed the actions of their seniors and caregivers. The oral health and hygiene practices of the children were also not found to be healthy in the religious schools where the caregivers had limited control over the children and children preferred to imitate their seniors or use cultural oral health practices.

Caregivers can adequately monitor children's oral health only if they have sufficient knowledge [19]. The oral health knowledge theme explains the understanding and perception of caregivers in this study. The knowledge perspective for cleaning the teeth was different. Inadequate, specific, nonspecific, and religiously oriented oral health knowledge was observed at these children’s care centres. Inadequate oral health knowledge refers to the belief of caregivers that oral health is not a health issue at all or that oral health is not an issue for children of this age group. This inadequate knowledge limits the caregivers from putting much emphasis on maintaining an appropriate oral health practice.

Oral health-specific knowledge was observed at some of the children’s care centres, where the caregivers showed their concern about good oral hygiene. Caregivers were found to have proficient knowledge and were concerned about oral health. Caregivers with religiously specific oral health knowledge recommended natural chewing sticks such as “Miswak”, which appeared as a popular and culturally embedded method at religious schools.

The criteria for oral health varied at different children’s care centres depending upon the availability of resources. The centres with moderate to low facilities considered oral health only concerned with mouth odour, and their choices were inadequate. This perspective was not considered as a category in this study but was an interesting input to be mentioned for future research. However, the privileged class centres were concerned even with the brand of the toothpaste and had adequate knowledge about oral health.

The hygienic practice of the caregivers varied across children's care centres and the availability of resources. Limited-resourced foster care centres were dependent on the donors and availability of oral hygienic supplies. Their approach to oral health was related to these factors and was not considered a matter of concern due to limited resources. Oral hygienic practices appeared as need-based, availability of dentifrices, oral health-oriented, compromised, and religiously preferred practices at children’s care centres. At the foster care homes and low-resource religious schools, limited emphasis was placed on maintaining children’s oral hygiene by the caregivers.

Toothpaste with dentifrices and *miswak* were common methods for cleaning the tooth at least once a day at boys' religious schools. Considering the low cost, simplicity and easy availability of Miswak, this natural method is widely used in many developing countries [20]. A strong affiliation exists between using Miswak and religious and cultural practices in Islamic countries [21]. The religious practice was found to use Miswak before every prayer, which accounts for five times per day in religious schools for similar beliefs.

In addition to these two methods, some children from the northern areas practised their regional norms and were using the babool chewing stick “*Dantan”*. The effectiveness of Dantan against antimicrobial activities in the mouth has been reported in some studies [22]. However, some oral health hazards associated with the use of Dantan were mentioned by the participants. This finding was another interesting perspective to explore oral hygiene and cultural settings in future research.

The health-seeking behaviour varied immensely between the two foster care centre settings and differed within the privileged level of the religious schools. Cultural remedies and self-medication by the recommendations from a dispenser or pharmacy assistant were the first treatment of choice at the low-privileged centres. Previous studies conducted on managing children's oral health by dispensers and pharmacy assistants showed a low personal self-efficacy compared to other staff [23, 24].

Seeking medical care was based on the personal preferences and convenience of the caregivers. Several oral health practices were used by the caregivers, such as self-medication, homeopathic medication, ethnomedicine, supplication-based practices, and nonhealthcare professional practices. Previous studies have suggested that integrated approaches to primary oral health by considering the local culture can be effective in improving oral health [25]. Restricted to merely local remedies can cause potential oral health hazards that need to be incorporated with proven scientific methods.

Girls’ religious schools had a stronger belief in spiritual healing to treat oral health issues with limited exposure to proper oral care. Low oral health literacy in seeking medical treatment among caregivers and the community can be a potential barrier for children to seek treatments [26]. It is also important to teach the caregivers about the potential oral health issues that can happen with children of this age group. Poor oral hygiene among these children can be a consequence of inadequate knowledge and the neglected behaviour of the caregivers, which can ultimately lead to oral health problems.

Interesting separate research can be conducted about any aspect of the health of those people; for example, *dandan saz* means “tooth makers”, but they are considered to be dental experts without any formal qualifications and approved dental equipment.

This study has the strength of being novel in Pakistan and focused on the oral health of children living in foster care centres and religious schools. This qualitative in-depth research provides an understanding of caregivers’ perceptions and practices related to childrens’ oral health. The ethnomethodological approach and FGDs explored caregivers’ perceptions and practices based on their personal reasoning in two different settings. This study had the limitations of a small sample size focusing on only a few centres in one city. The findings of this study recommend using a mixed-method approach to evaluate the thematic findings with the clinical examination of the children. The awareness of caregivers can be improved by arranging seminars, training sessions, and workshops to train them to work with under-care children at foster care centres and religious or other boarding schools.

## Conclusion

This qualitative study concludes that the oral health of the children at care centres and religious schools depends upon the personal reasoning and preexisting religio-cultural knowledge of the caregivers rather than on specialized oral health-oriented approaches. The caregivers at foster care centres were more involved in supervising the children to maintain oral hygiene and oral health compared to religious schools.

## Supplementary Information

**Additional file 1**: Interview guide.

## Data Availability

This is a qualitative study. For privacy reasons study participants, these data cannot be made publicly available. However, data are available from the corresponding author upon reasonable request.

## Abbreviations

COREQ: Consolidated criteria for reporting a qualitative research
FG: Focus group
FGD: Focus group discussion
