# Supplementary material for: Oral health perceptions and practices of caregivers at children’s religious schools and foster care centers: a qualitative exploratory study in Lahore, Pakistan
Source: BMC Oral Health. 2022 Dec 24;22:641. doi: 10.1186/s12903-022-02687-0 (PMC9789729; doi:10.1186/s12903-022-02687-0)
Supplement: Supplementary file 1 — Additional file 1: Interview guide. [file 12903_2022_2687_MOESM1_ESM.pdf]

FGD interview guide for caregivers at foster care centers and religious schools

Introduction:

- Demographic information
- Time at foster care centers/ religious schools
- Roles other than the caregiver
- Administrative Structure of the
- Reasons for the children to be in the foster care centers/in the religious schools, other than education?
- If some of the children's mothers live with them in the foster care centers?

Perceptions of the oral health

- What do you know about oral health? (Origin of the understanding, if cultural or religious)
- Do the children know about oral health at the time of admission?
- Their socioeconomic background?
- Overall health and oral health condition of the children?

Probing questions

Importance of the oral health

- Are any specific activities about oral health?
- The eating habits of the children? Sweets, fizzy drinks, smoking.
- Are there any oral health-focused restrictions?

Probing questions

Oral hygiene practices

- If they supervise children's oral hygiene?
- Why? Who? How? When/ how many times in a day?
- Type of densifiers used. Any specific reasons? How long it lasts?

Probing questions

Oral health practices

- Do children suffer from dental aches?
- How do the children get oral health treatment?
- Reasons, if that is effective? Adequate. If they know about dental treatment.

Probing questions

Anything else you want to add/say?
